# Dentin, Dentin Graft, and Bone Graft: Microscopic and Spectroscopic Analysis

**DOI:** 10.3390/jfb14050272

**Published:** 2023-05-13

**Authors:** Elio Minetti, Andrea Palermo, Giuseppina Malcangi, Alessio Danilo Inchingolo, Antonio Mancini, Gianna Dipalma, Francesco Inchingolo, Assunta Patano, Angelo Michele Inchingolo

**Affiliations:** 1 Department of Biomedical, Surgical, Dental Science, University of Milan, 20161 Milan, Italy; elio.minetti@unimi.it; 2 College of Medicine and Dentistry, Birmingham B4 6BN, UK; andrea.palermo2004@libero.it; 3 Department of Interdisciplinary Medicine, University of Bari “Aldo Moro”, 70124 Bari, Italy; giuseppinamalcangi@libero.it (G.M.); ad.inchingolo@libero.it (A.D.I.); dr.antonio.mancini@gmail.com (A.M.); angeloinchingolo@gmail.com (A.M.I.)

**Keywords:** autologous tooth graft, bone, bone substitute material, bone tissue regeneration, demineralization, dentin, Tooth Transformer

## Abstract

Background: The use of the human dentin matrix could serve as an alternative to autologous, allogenic, and xenogeneic bone grafts. Since 1967, when the osteoinductive characteristics of autogenous demineralized dentin matrix were revealed, autologous tooth grafts have been advocated. The tooth is very similar to the bone and contains many growth factors. The purpose of the present study is to evaluate the similarities and differences between the three samples (dentin, demineralized dentin, and alveolar cortical bone) with the aim of demonstrating that the demineralized dentin can be considered in regenerative surgery as an alternative to the autologous bone. Methods: This in vitro study analyzed the biochemical characterizations of 11 dentin granules (Group A), 11 demineralized using the Tooth Transformer (Group B), and dentin granules and 11 cortical bone granules (Group C) using scanning electron microscopy (SEM) and energy dispersive spectroscopy (EDS) to evaluate mineral content. Atomic percentages of C (carbon), O (oxygen), Ca (calcium), and P (phosphorus) were individually analyzed and compared by the statistical t-test. Results: The significant *p*-value (*p* < 0.05) between group A and group C indicated that these two groups were not significantly similar, while the non-significant result (*p* > 0.05) obtained between group B and group C indicated that these two groups are similar. Conclusions: The findings support that the hypothesis that the demineralization process can lead to the dentin being remarkably similar to the natural bone in terms of their surface chemical composition. The demineralized dentin can therefore be considered an alternative to the autologous bone in regenerative surgery.

## 1. Introduction

The tooth grafting process has been introduced by Urist et al. more than 50 years ago, when they uncovered the osteoinductive properties of the demineralized dentin matrix [1,2]. Fundamental growth agents for bone regeneration can be found in both the bone and the dentin matrix. These biological materials have been suggested as potential graft materials by several authors. Bone is composed of 61% inorganic mineral, and 39% organic substance and water [3,4,5,6].

The organic part (39%) is made up of 90% collagen type I, with the remaining 10% being made up of non-collagen proteins produced by the bone cells. Ten percent of the total organic content is then incorporated into the bone matrix during the process of osteosynthesis [7].

The Ca/P molar ratio of bone apatite nanocrystals differs from the stoichiometric hydroxyapatite ratio of 1.67 due to a variety of substitutions and vacancies. The crystals in the bone (low crystalline calcium phosphate) are nanosized, measuring 20–60 nm in length and 5–20 nm in breadth [8,9,10].

Some of the important non-collagenous proteins are osteocalcin (Gla proteins), osteopontin, bone sialoproteins, and osteonectin. The bone matrix also contains proteoglycans, bone morphogenetic proteins (BMPs), and a variety of growth factors, including platelet-derived growth factor (PDGF), fibroblast growth factor (FGF), and insulin-like growth factors (IGF). Moreover, lysyl oxidase and tyrosine-rich acidic matrix proteins (TRAMP) are further important components of the demineralized bone matrix. Growth factors are fixed and stored in enormous concentrations in the mineralized matrix of bone. According to this hypothesis, growth factors such as IGF-II are deposited for a period of time before being released in a bioactive form by osteoblast bone resorption to act on pre-osteoblasts and mature osteoblasts, thereby allowing for the site-specific replacement of bone lost to resorption [2,11,12,13].

Dentin and cement are two different specialized forms of bone. The dentin is harder than the compact bone, and is made up of organic matrix (28%) and inorganic matrix (72%) [14], and these crystals are the same size for dentin and bone [15,16].

Dentin consists of 35% organic matter (90% collagen type 1, with the remaining 10% non-collagenous proteins including BMPs), water, and 65% inorganic material (hydroxyapatite in high crystalline calcium phosphate). The majority of dentin is made up of proteins that are found in both the dentin and bone [17,18].

Proteins common in both bone and dentin include: collagen types I, III, and V, bone sialoprotein (BSP), osteopontin (OPN), dentin matrix protein-1 (DMP-1), osteocalcin (OC), and osteonectin (ON) [17,19].

The organic substance (making up 10% of the 35% organic matter) comprises collagenous fibrils embedded in mucopolysaccharide ground substance. The main kind of collagen found in dentin is type I collagen. The matrix is synthesized by odontoblasts and is a rich source of growth factors and contains bioactive molecules required for dentinogenesis. These molecules are released in the presence of bacterial acids or certain dental materials in the case of caries or restorative treatments, causing dentin regeneration and repair [17,20].

Proteoglycans such as chondroitin sulfates, decorin, and biglycan; glycoproteins including dentin sialoprotein (DSP), osteonectin, and osteopontin; phosphoproteins such as dentin phosphoprotein (DPP), and gamma carboxy-glutamate containing proteins (GLA-proteins), and phospholipids, are important constituents of the ground substance. Dentin matrix and bone protein are similar; however, dentin sialoprotein and dentin phosphoprotein are uniquely found in dentin. TGF, FGF, IGFs, BMPs, epidermal growth factor (EGF), PDGF, placenta growth factor (PLGF), vascular endothelial growth factor (VEGF), and angiogenic growth factor (AGF) are all found in the matrix. These matrix components play critical roles in dentin mineralization, and include various growth factors including transforming growth factor-b (TGF-b1), IGF, BMPs, and several angiogenic growth factors [21,22,23,24].

Dentin represents an efficient source of BMPs, bioactive growth factors (GFs), and transforming growth factor-B (TGF-B), which all play a role in bone repair processes [25].

The first to theorize the presence of GFs in dentin was Urist in 1971, who stated that the BMPs, which stimulate bone formation, are found in dentin, as are non-collagenous proteins such as osteocalcin, osteonectin, and dentin phosphoprotein [26].

According to experts, the demineralization process provides superior bone augmentation compared to non-demineralized dentin [27,28].

A tooth graft without any treatment is contaminated and is not safe to use it in surgical procedure [29], but recently an innovative medical device was introduced to the market that is able to automatically clean and demineralize, as well as obtain suitable tooth graft materials starting from the whole tooth of the patient. In vitro investigations on the graft material generated by this innovative technology showed that the demineralization process increases BMP-2 bioavailability [30,31].

BMP-2 is important in the control of odontoblast differentiation and dentin production [32,33,34,35]. Nampo et al. in 2010 analyzed all the growth factors presence in both the dentin and bone [36].

BMP-2 significantly increases bone growth in the demineralized dentin matrix (DDM) carrier system [4,19,30,37].

The mineral component is composed of hydroxyapatite crystals, with carbonate content and a lower Ca/P ratio than the pure hydroxyapatite. The inorganic component consists of hydroxyapatite, as in bone. The crystals are plate-shaped and much smaller than the hydroxyapatite crystals in enamel but are ten times bigger than the bone. The incremental lines of von Ebner reflect the daily rhythmic, recurrent deposition of the dentin matrix. The course of the lines indicates the growth pattern of the dentin. The daily deposition is approximately 4 µm. Dentin is similarly mineralized in a 12 h cycle [38,39,40,41].

Crystals of hydroxyapatite (HA) are present in the bone and in the dentin in the shape of plates or needles. These crystals are round long, wide, and thick (Table 1). The major mineral component of teeth and bones is hydroxyapatite (Ca10(PO4)62(OH). The structure of bone contains around 65 wt.% hydroxyapatite, a needle-shaped compound with a length of 20–60 nm and a width of 5–20 nm that is responsible for stiffness and strength. Using X-ray diffraction (XRD), the average size of hydroxyapatite crystallites in dentin was estimated. The crystallites were found to be in the form of flattened plates, 80 + −12 nm in length, 3–4 nm in thickness, and 40 + −10 nm in width [42,43].

The aim of this study was to compare the mineral content, using scanning electron microscopy (SEM) and energy dispersive spectroscopy (EDS), of the dentin, the demineralized dentin, and the cortical bone. We aimed to analyze these three samples to evaluate both the similarities and differences with a particular interest on demineralized dentine vs. bone to understand whether the demineralization treatment makes the demineralized dentin similar to the bone.

## 2. Materials and Methods

Twenty-two dentin samples and eleven bone samples were collected from patients according to the Declaration of Helsinki guidelines and was approved by the local Ethical Committee for Biomedical Research of Chieti and Pescara (Prot. N. 1869/21.03.2019). The bone samples, with signed patient informed consent, were taken from broken bone residues remaining attached to the extracted teeth roots or collected at the time of implants insertion. The study included patients over 18 years of age who needed a tooth extraction treatment, in good health condition (ASA-1 and ASA-2) without any alteration of bone metabolism condition and without any pharmacological use, and who were able to undergo dental surgical and restorative procedures. Tooth extractions were needed for trauma, caries, or periodontal diseases.

The dentin samples were randomly subdivided into two groups. group A (dentin without demineralization), and group B (dentin with demineralization). Following the Minetti’s group indications, teeth with root canal treatments were also utilized as in these studies it was shown that there were no significant differences in results [3].

The demineralization process was made using the innovative medical device Tooth Transformer^®^ (TT, from Tooth Transformer^®^ Srl, via Washington 59 20146, Milan, Italy), which can collect acceptable tooth graft materials beginning with the entire tooth. This technology ensures thoroughly automated cleaning, grinding, and demineralizing procedures, with no errors caused by human intervention. The Tooth Transformer is a cutting-edge tissue engineering device, as it can quickly process and turn a removed tooth into clinically viable bone graft material [44].

A piezoelectric tool (Mectron, Carasco (GE), Italy) was used to clean the entire excised tooth of remaining calculus. The root surface was polished with a diamond drill (ref.6855 Dentsply Maillefer, Ballaigues, Switzerland), and the tooth’s filling materials (guttapercha, composite, etc.) were carefully removed. The tooth was then chopped into little pieces and placed into the device’s mill. The single-use unit was opened, and a little box containing disposable liquids was inserted at the correct position in the device (indicated by arrows). The device was started using the general switch button once all the components were installed and the machine’s cover was closed, and the demineralized dentin graft was ready. The bone was the third group (group C).

The bone samples (eleven samples from the lower molar) were collected at the time of the implant’s insertion. After a signed consensus from the patient, a 3 mm trephine bur (MEISINGER USA, L.L.C. 10150 E. Easter Avenue Centennial, CO 80112, USA) was used to prepare the implant site, and then the dedicated implants drills were used under extensive irrigation with saline solution. The bone removed to create a surgical implant alveolus was collected. The sample was then washed with physiological solution to carefully remove any blood residues or other tissue frustules and was immediately inserted into the freshly prepared fresh fixative solution and stored away from light (10% neutral buffered formalin) in a flask of at least 10 cc of hermetic sealant volume without bubbles.

Each sample of each group was analyzed using a scanning electron microscopy (SEM) device.

An environmental scanning electron microscope (ESEM Zeiss EVO50, Carl Zeiss, Milan, Italy) linked to a secondary electron detector for energy dispersive X-ray (Carl Zeiss, Milan, Italy) nalysis was used to analyze the surface morphology of Group A-B-C sample particles.

Following a 2 h fixation with 1.5% (*v*/*v*) glutaraldehyde fixation and dehydration in progressive ethanol, samples were gold-sputtered, mounted on scanning electron microscopy (SEM) stubs, and then evaluated with a 15 kV acceleration voltage. SEM pictures were captured at a magnification of 5000. All the particles from each group were examined with EDS to analyze the surface composition (atomic percentages of carbon, oxygen, phosphorus, and calcium).

## 3. Results

A total of 33 analysis were performed: 22 dentin samples were analyzed from 22 different extracted teeth, 11 bone biopsies of different sites from 11 subjects (6 male and 5 women) aged 53.8 + −6.56 were performed.

**Figure 1:**
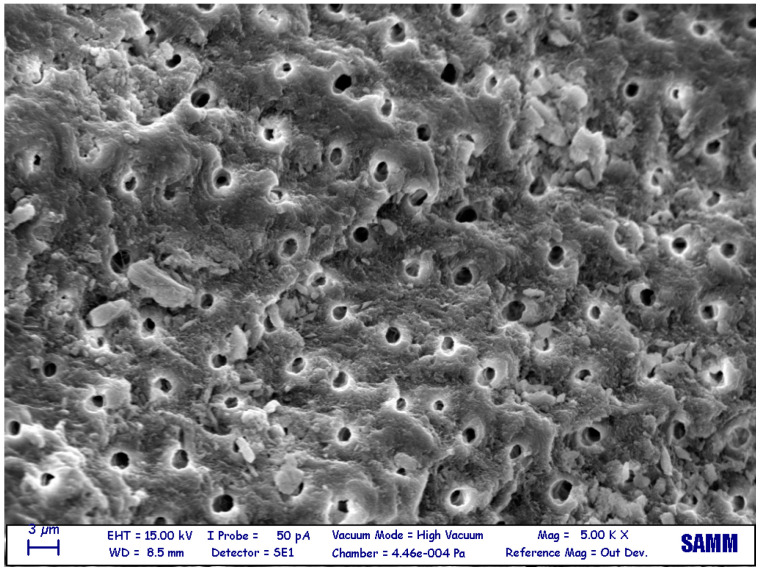
Electron microscopy (5000×) surface of a non-demineralized dentin granule. The surface is rough, dirty, and full of debris, and the dental tubules are not clean or closed.

**Figure 2:**
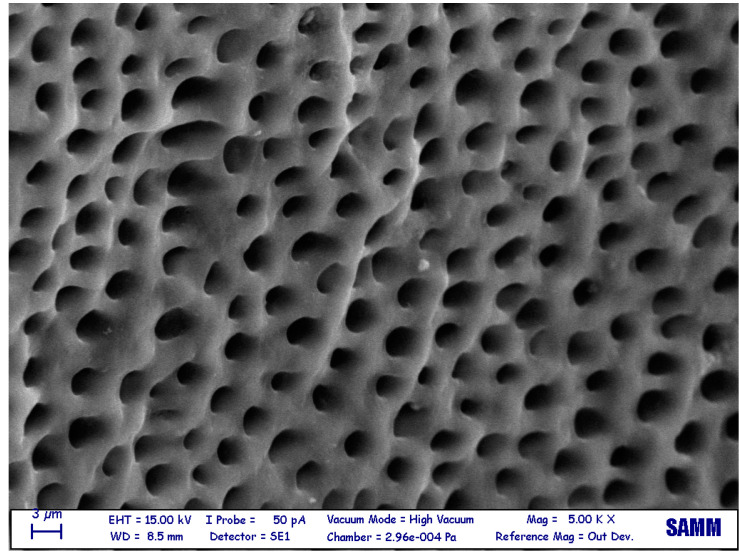
Electron microscopy (5000×) surface of a demineralized dentin after the Tooth Transformer treatment. The surface is cleaner and smoother than non-demineralized dentin.

**Figure 3:**
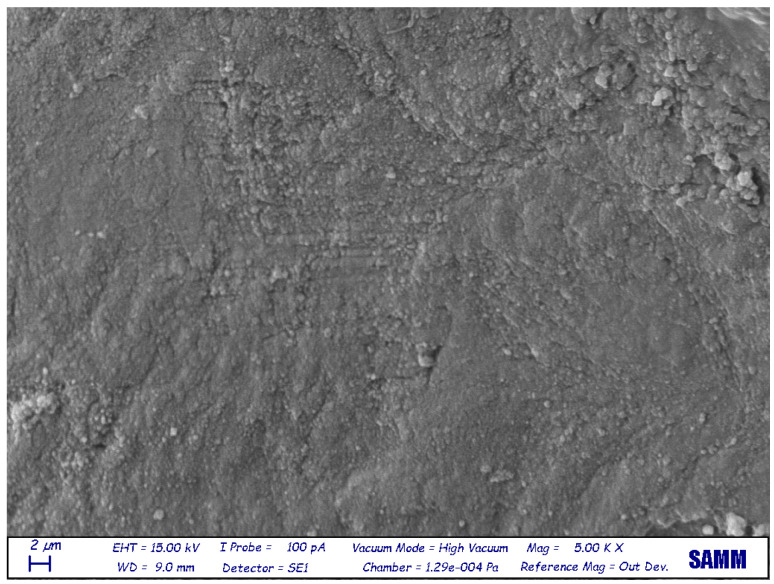
Electron microscopy (5000×) surface of a bone sample. The surface is smooth, flat, and free of irregularities and dental tubules.

The sample surfaces were found to be different between the various groups as shown in the pictures (Figure 1, Figure 2 and Figure 3).

The summary of the results of the EDS analysis are presented in Table 2 and Table 3. Statistical analysis was conducted between group A (non-treated dentin) vs. group C (bone), and between group B (demineralized dentin) and group C (bone). No statistical differences between group B (demineralized dentin) and group C (bone) were found. There was, however, a statistical difference observed between Group A (non-treated dentin) and group C (bone).

## 4. Discussion

Vertebrates originate from three germ layers: the ectoderm, mesoderm, and endoderm, as well as neural crest cells, which arise from the neural tube fusion region, and both the teeth and the alveolar bone are formed by neural crest cells and contain type I collagen. The maxillofacial bones (except for the occipital, sphenoid, temporal, and ethmoid bones), cartilage, teeth, and nerve and glial cells are all produced from the neural crest [36,45].

There are a lot of similarities displayed between the information presented in Table 4 and Table 5. At the tissue scale, the bone and dentin tissues are different, but at the ultrastructural scale both are made with the same constituents.

The primary morphologic distinction between the bone and the dentin is that certain osteoblasts occur on the surface of bone, and when one of these cells becomes encased within its matrix, it is then referred to as an osteocyte. Meanwhile, the cell bodies of odontoblasts remain external to dentin [46]. Dentin has the same chemical composition as bone and is regarded a suitable bone substitute. The mineralization of both bone and dentin is conducted in the same way [47,48,49].

The mineralization process includes proteins deposited into both matrices, and the impact of this process is the presence of the same proteins embedded.

In general, biomineralization consists of the process by which cells organize mineral deposition. It represents the cell-mediated process in which HA is deposited into the extracellular matrix (ECM) of the skeletal structures present in vertebrates. ECM structural molecules, along with several enzymes, direct mineral salt entrance and fixing exclusively in the bone and mineralized tooth tissues [49].

Table 6 describes the proteins that can be found in the bone and dentin.

The bone graft was used because it is the same tissue. Socket grafting has been demonstrated to be more effective in terms of bone quantity and quality than excision alone [50].

The autologous bone graft contains osteogenic properties (marrow-derived osteoblastic cells as well as preosteoblastic precursor cells), osteoinductive properties (non-collagenous bone matrix proteins, including growth factors), and osteoconductive properties (bone mineral and collagen). Being an autologous graft, there is no risk of disease transmission and perfect histocompatibility. However, there are some disadvantages to using autogenous bone, such as insufficient graft material, the possibility of significant postsurgical morbidity at the donor site (e.g., rib, fibula, or iliac crest), such as infection, pain, hemorrhage, muscle weakness, and nerve injury, increased surgical time and blood loss, and additional cost (Table 6) [51].

While deciding where to collect bones, the amount and quality of donor bone sites should also be evaluated (Table 7) [52].

There are certain disadvantages to using autogenous bone, such as insufficient graft material, due to the need for an additional surgical site. Therefore, the possibility to utilize different materials was evaluated. In particular, the dentin, as shown before, has the same properties of the bone and was previously considered as an efficient graft. Using an autologous extracted tooth there is a complete histocompatibility and no opportunity for disease transmission.

The guided bone regeneration (GBR) healing phases are similar between the dentin and the bone because they are remarkably similar autologous tissues.

Phase 1

4–6 WEEKS

During the first week after surgery, the graft is surrounded by a slew of inflammatory cells such as lymphocytes, plasma cells, osteoclasts, mononuclear cells, and polynuclear cells. There is also some fibrous tissue present. In the second week, fibrous granulation tissue predominates at the recipient location, and osteoclastic activity increases. Invading macrophages destroy the necrotic tissue within the graft’s haversian canals, resulting in the production of intracellular byproducts that, combined with the recipient site’s low oxygen tension and pH, function as a chemoattractant to host undifferentiated stem cells. Mesenchymal cells begin to develop into osteogenic cells when exposed to osteoinductive stimuli. The first phase of bleeding, inflammation, revascularization, and osteoinduction proceeds as a continuous process, with active bone production and resorption occurring within four weeks of implantation. Cancellous autografts are then integrated into a necrotic bed by the creation of new bone. As a result, the construct’s mechanical qualities are initially strengthened. The mechanical strength of the graft–host interface eventually returns as necrotic bone is resorbed and replaced [10,14].

4–6 WEEKS

Formation of the clot and migration of the vascular structures in the bone walls around the defect. The deposition of the osteoid bone starts. The cellular and molecular cascades involve cell migration from the surrounding tissue. The cells secrete factors that are essential for bone formation and remodeling. This favors the mature remodeled bone development in the underlying defect by activating the activity of osteoblasts and osteoclasts [14].

Phase 2

8–12 WEEKS

Maturation of the osteoid bone occurs and cortical bone development begins. The marrow bone will be mineralized from the osteoblasts. New cortical bone will begin to form on its periphery [14].

Phase 3

12–16 WEEKS

Maturation of the cortical bone occurs, and the remodeling of the marrow and cortical bones begin. Viewing near the membrane can permit observations of the newly modeled cortical bone [53,54].

Recently, there has been a lot of interest in autologous bone-like materials as potential substrates for bone regeneration of alveolar lesions. More particularly, the utilization of tooth-derived materials has recently piqued the curiosity of many individuals due to the natural abundance of teeth that are pulled every day and abandoned as trash [3,27].

In histology books, the dentin and the tooth cementum are two specialized forms of bone tissue. Dentin is a special form of bone tissue that is harder than compact bone [14].

Physically and chemically, dentin resembles bone. The final components of the tooth and bone are the same: collagen, hydroxyapatite, and non-collagenic proteins. In fact, dentin, in the form of native dentin and dentin derivatives, such as demineralized and deproteinized dentin have thus been used as graft materials in bone repair processes. Some experimental evidence issued from a critical literature review highlights the role of demineralized dentin matrix in stimulating osteodifferentiation in vitro and, in increasing in vivo osteoinduction [46,55,56,57,58].

The presence of growth factors, and the building of both the bone and tooth with collagen type 1, and the same inorganic molecule of hydroxyapatite Ca5(PO4)3(OH), suggests to presume possible, after following the correct procedure about disinfection and cleaning to use the tooth as a bone graft [22,59,60].

The treatment submitted from the tooth will help determine the minor or major presence of growth factors. In fact, studies from Bono and Candiani reveal the different GFs present depending on the different liquids [30].

If the dentin is considered similar to the bone, the demineralized dentin should therefore be considered very similar to the bone. The limitation of this in vitro test is the limited numerosity, and it would be far more optimal to obtain these results in vivo. These in vitro tests conclude that there are no statistical differences present between group B and Group C, and that the ratio between Ca/P is remarkably similar.

## 5. Conclusions

The treated dentin components values are remarkably similar to the bone component values. Tests were conducted to demonstrate that the activity carried out by the device (automated treatment with HCL and H2O2, along with a different temperature and UVA) allows a modification of the chemical compositions of the dentin to make it statistically similar to bone tissue more than the dentin without treatment. This clearly implies that the tooth can be used by means of a dedicated medical device that guaranteed the treatment in an automatic, repeatable way, and as an alternative to the use of the autologous bone in regenerative treatment.

The autologous ooth has already been used to make sinus lifts, GBR, and alveolar ridge preservation socket preservation.

More studies and different numerosities are necessary to better understand the influence of the treatment in tooth grafting.

## Figures and Tables

**Table 1:** Bone and dentin HA crystal dimensions.

HA Crystals	Bone	Dentin
Length	20–60 nm	80 ± 12 (nm)
Width	5–20 nm	40 ± 10 nm
Thickness	1, 2/3 nm	3–4 nm

**Table 2:** Comparison between group A and group C. Eleven samples were analyzed by elemental analysis, and atomic percentages of C (carbon), O (oxygen), Ca (calcium), and P (phosphorus) were individually analyzed and compared. The *p*-value was indicated for each comparison, and all comparisons were found to be statistically different. Analyze: *p* > 0.05—no statistical significance value. If *p* < 0.05—the hypothesis is wrong.

Group A vs. Group C												
Dataset	C	Non-treated dentin	Bone	O	Non-treated dentin	Bone	Ca	Non-treated dentin	Bone	P	Non-treated dentin	Bone
Sample size		11	11		11	11		11	11		11	11
Average		24.0200	44.9710		49.9800	13.3540		16.5600	9.9260		8.5900	4.0280
Standard deviation		4.3500	11.7680		4.6500	1.7400		6.1200	6.7470		1.9100	2.7700
T		5.5384			24.4668			2.4154			4.4969	
Degree of freedom		20			20			20			20	
*p*-value		0.00002			0.00000000002			0.0254			0.0002	

**Table 3:** Comparison between group B and group C. Eleven samples were analyzed by elemental analysis, and atomic percentages of C (carbon), O (oxygen), Ca (calcium), and P (phosphorus) were individually analyzed and compared. The *p*-value was indicated for each comparison, and all are without any statistical differences. Analyze: *p* > 0.05—no statistical significance value. If *p* < 0.05—the hypothesis is wrong.

Group B vs. Group C												
Dataset	C	Treated dentin	Bone	O	Treated dentin	Bone	Ca	Treated dentin	Bone	P	Treated dentin	Bone
Sample size		11	11		11	11		11	11		11	11
Average		60.0200	44.9710		26.0600	13.3540		8.5900	9.9260		5.0400	4.0280
Standard deviation		3.7900	11.7680		3.0600	1.7400		1.3700	6.7470		0.6000	2.7700
T		4.0371			11.9715			0.6436			1.1842	
Degree of freedom		20			20			20			20	
*p*-value		0.0006			0.00002			0.5271			0.2502	

**Table 4:** Bone and dentin differences at different scales.

	Bone	Dentin
Tissue scale (millimeters to micrometers)	Bone is an organic matrix of connective tissue composed of cells, fibers, and inorganic matrix ground substance. The cells control the initial production of the mineralized tissue.	Teeth are composed of cells, an organic matrix, and an inorganic matrix.
Microstructure scale (micrometers)	Individual struts (trabeculae) present in the marrow connecting the bone structure, thin plates (lamellae) in the cortical bone, and bone developed around blood veins are all structural units of bone (termed osteons).	Dentinal tubules and the intratubular dentin that surrounds the dentin-forming odontoblasts are structural units of the tooth.
Ultrastructural scale (nanometers)	Tissue components are distinct in the mineral crystals and the organic matrix. The bone’s organic matrix mostly comprises of a fibrous protein, collagen, and trace amounts of other non-collagenous proteins.	Collagen is the primary organic constituent of dentin and cementum. However, there is no collagen present in enamel. An equivalent of the mineral hydroxyapatite is the mineral that reinforces dentin matrices and is also a major constituent of enamel.

**Table 5:** Different mineralization phases between the bone and dentin.

Mineralization Phase	Bone	Dentin
First phase	Osteoblasts secrete organic matrix (in particular collagen and non-collagenic proteins) and bone vesicle matrix	Odontoblasts secrete collagen and non-collagenic proteins.
Second phase	Also termed the vesicular phase, vesicles—which have accumulated calcium and phosphorus—begin to nucleate the calcium and phosphorus salts. The crystalline structures grow due to the entry of phosphorus and calcium ions, resulting in the breakage of the vesicle membrane.	Nucleation of vesicles -which have accumulated calcium and phosphorus. The osteoblast elongates a cytoplasmatic process into the dentinal tubules.
Third phase	Termed the fibrillar phase, the hydroxyapatite crystals further increase in the bone extracellular matrix and form the so-called matrix nodules that are associated with the organic matrix of the osteoid substance, in particular with collagen fibrils. It is precisely on the collagen fibers that the hydroxyapatite crystals are deposited, and therefore we speak, in fact, of “collagen mineralization”.	Growth of the crystals occurs. The crystals linked to the collagen fibrils are arranged in rows that conform to the 64 nm striation pattern, with their long axes paralleling the fibril long axes. The progress of general calcification is gradual. Dentin apatite crystals are similar to those found in the bone and cementum, and are 300 times smaller in size than those made in the enamel.

**Table 6:** List of proteins present in the bone and dentin.

Main Bone Proteins	Main Dentin Proteins
GLA protein	GLA protein
OPN	OPN
Osteonectin	Cbfa 1 RUNX2
Proteoglycans	BMP
BMP	IGI I and IGF II
PDGF	TGF-b 19
FGF	DSP
IGF	DGP, DPP
Lysyl oxidase	Type I, III, V collagen
TRAMP	
BSP	
Type I, III, V collagen	

**Table 7:** The quantity of donor bone sites calculated from Somsak/Rajesh analysis.

Donor Sites	Size of Corticocancellous Block	Volume
Symphysis	20.9 × 9.9 × 6.9 mm^3^	4.71
Ascending ramus	37.6 × 33.17 × 22.48 × 9.15 mm^3^	2.36
Lateral ramus	1.3 cm × 3 cm^3^	Not applicable
Coronoid process	18 × 17 × 5 mm^3^	Not applicable
Zygomatic buttress	1.5 × 2.0 mm^3^	Not applicable

## Data Availability

Not applicable.

## Abbreviations

PDGF	Platelet-derived growth factor
FGF	Fibroblast growth factor
IGF	Insulin-like growth factor
TRAMP	Tyrosine-rich acidic matrix protein
BMP	Bone morphogenetic protein
BSP	Bone sialoprotein
OPN	Osteopontin
DMP-1	Dentin matrix protein-1
OC	Osteocalcin
ON	Osteonectin
ELISA	Enzyme-linked immunosorbent assay
TGF	Transforming growth factor
DSP	Dentin sialoprotein
DPP	Dentin phosphoprotein
GLA-proteins	Gamma-carboxyglutamate-containing proteins
EGF	Endothelial growth factor
PLGF	Placenta growth factor
VEGF	Vascular endothelial growth factor
AGF	Angiogenic growth factor
GF	Growth factor
